# BN-BacArena: Bayesian network extension of BacArena for the dynamic simulation of microbial communities

**DOI:** 10.1093/bioinformatics/btae266

**Published:** 2024-04-30

**Authors:** Telmo Blasco, Francesco Balzerani, Luis V Valcárcel, Pedro Larrañaga, Concha Bielza, María Pilar Francino, José Ángel Rufián-Henares, Francisco J Planes, Sergio Pérez-Burillo

**Affiliations:** Department of Biomedical Engineering and Sciences, Tecnun School of Engineering, University of Navarra, San Sebastián 20018, Spain; Department of Biomedical Engineering and Sciences, Tecnun School of Engineering, University of Navarra, San Sebastián 20018, Spain; Department of Biomedical Engineering and Sciences, Tecnun School of Engineering, University of Navarra, San Sebastián 20018, Spain; Biomedical Engineering Center, Campus Universitario, University of Navarra, Pamplona, Navarra 31009, Spain; Instituto de Ciencia de los Datos e Inteligencia Artificial (DATAI), Campus Universitario, University of Navarra, Pamplona 31080, Spain; Departamento de Inteligencia Artificial, Universidad Politécnica de Madrid, Madrid 28660, Spain; Departamento de Inteligencia Artificial, Universidad Politécnica de Madrid, Madrid 28660, Spain; Area de Genómica y Salud, Fundación para el Fomento de la Investigación Sanitaria y Biomédica de la Comunitat Valenciana-Salud Pública, Valencia 46020, Spain; CIBER en Epidemiología y Salud Pública, Instituto de Salud Carlos III, Madrid 28029, Spain; Departamento de Nutrición y Bromatología, Centro de Investigación Biomédica, Instituto de Nutrición y Tecnología de los Alimentos, Universidad de Granada, Granada 18016, Spain; Instituto de Investigación Biosanitaria ibs. GRANADA, Universidad de Granada, Granada 18012, Spain; Department of Biomedical Engineering and Sciences, Tecnun School of Engineering, University of Navarra, San Sebastián 20018, Spain; Biomedical Engineering Center, Campus Universitario, University of Navarra, Pamplona, Navarra 31009, Spain; Instituto de Ciencia de los Datos e Inteligencia Artificial (DATAI), Campus Universitario, University of Navarra, Pamplona 31080, Spain; Department of Biomedical Engineering and Sciences, Tecnun School of Engineering, University of Navarra, San Sebastián 20018, Spain

## Abstract

**Motivation:**

Simulating gut microbial dynamics is extremely challenging. Several computational tools, notably the widely used BacArena, enable modeling of dynamic changes in the microbial environment. These methods, however, do not comprehensively account for microbe–microbe stimulant or inhibitory effects or for nutrient–microbe inhibitory effects, typically observed in different compounds present in the daily diet.

**Results:**

Here, we present BN-BacArena, an extension of BacArena consisting on the incorporation within the native computational framework of a Bayesian network model that accounts for microbe–microbe and nutrient–microbe interactions. Using *in vitro* experiments, 16S rRNA gene sequencing data and nutritional composition of 55 foods, the output Bayesian network showed 23 significant nutrient–bacteria interactions, suggesting the importance of compounds such as polyols, ascorbic acid, polyphenols and other phytochemicals, and 40 bacteria–bacteria significant relationships. With test data, BN-BacArena demonstrates a statistically significant improvement over BacArena to predict the time-dependent relative abundance of bacterial species involved in the gut microbiota upon different nutritional interventions. As a result, BN-BacArena opens new avenues for the dynamic modeling and simulation of the human gut microbiota metabolism.

**Availability and implementation:**

MATLAB and R code are available in https://github.com/PlanesLab/BN-BacArena

## 1 Introduction

There is an ever-increasing body of literature that supports a key relationship between gut microbiota and different aspects of human health (Gentile and Weir 2018). As a result, huge efforts have been made to elucidate the inner workings of bacteria-host interactions. The tremendous complexity of these interactions has triggered the development of different computational models to explain them, leading to testable hypothesis that can be validated via *in vitro* or interventional studies (Fuertes *et al.* 2019).

Particularly, the use of genome-scale metabolic models of bacterial species present in the gut has built up during the last years and high-quality repositories are now available for a huge number of species (Machado *et al.* 2018), including AGREDA (Blasco *et al.* 2021) and AGORA (Magnúsdóttir *et al.* 2016). These reconstructions have enabled the simulation of the metabolic capabilities of the human gut microbiota for very different scenarios, mainly based on constraint-based bacterial community models (Li *et al.* 2023). Though very promising, these models are not able to explore dynamic changes in either microbial abundance or metabolite production. This is of critical importance when it comes to evaluating the interaction diet-gut microbes because diet itself is dynamic as is the ecological environment that comprises the gut. There are some computational tools that allow modeling dynamic changes in the microbial environment, such as Comets (Dukovski *et al.* 2021) or BacArena (Bauer *et al.* 2017). They combine constraint-based and agent-based modeling to update metabolite input and bacterial abundance at each iteration and simulate time evolution. However, these tools do not comprehensively account for microbe–microbe stimulant or inhibitory effects or nutrient–microbe inhibitory effects, globally observed in compounds present in the daily diet (Poirier *et al.* 2016).

Here, we present BN-BacArena, an extension of BacArena that incorporates in the native computational framework a Bayesian network model that integrates microbe–microbe and nutrient–microbe interactions. The Bayesian network was built using 16S rRNA gene sequencing data obtained from gut microbial *in vitro* fermentation of 44 foods and their associated nutritional composition. Simulated bacterial growth obtained by BN-BacArena was then validated on a set of 11 *in vitro* fermented foods. BN-BacArena shows that the inclusion of our Bayesian network model significantly improves the accuracy for predicting bacterial abundances in the gut microbiota. BN-BacArena was developed in R by extending native functions of BacArena. However, we also translated BN-BacArena into MATLAB for integration with the COBRA Toolbox (Heirendt *et al.* 2019), a popular platform for constraint-based reconstruction and analysis of metabolic networks, which will increase the accessibility and use in the Systems Biology community.

## 2 Materials and methods

### 2.1 *In vitro* digestion and gut microbial *in vitro* fermentation

Food samples were *in vitro* digested following the protocol described by Brodkorb *et al.* (2019). Five grams of each sample were weighed (in triplicate) into 50 ml centrifugation tubes. Five milliliters of simulated salivary fluid with 150 U/ml of α-amylase were added into the tube carrying the sample and kept at 37°C for 2 min. Then, 10 ml of simulated gastric fluid with 4000 U/ml of gastric pepsin was added to the mix, the pH was lowered to 3, and kept at 37°C for 2 h. Finally, 20 ml of simulated intestinal fluid with 200 U/ml of pancreatin and 20 mM bile salts were added into the tube, the pH increased to 7 and kept at 37°C for 2 h. Enzyme activity was halted by immersion in ice for 15 min. Tubes were centrifuged, the supernatant (fraction available for absorption at the small intestine) collected and the pellet (fraction not digested that would reach the colon) used for *in vitro* fermentation.

*In vitro* fermentation was carried out following the protocol described by Pérez-Burillo *et al.* (2021). Fecal samples were collected from three healthy donors (BMI = 21.3–23.8 and they had not taken antibiotics in the last 3 months). Fecal material was pooled to account for interindividual variability. *In vitro* fermentation was carried out at 37°C for 20 h, under oscillation. For this purpose, 0.5 grams of the pellet obtained after *in vitro* gastrointestinal digestion were used, as well as 10% of the supernatant. Fermentation medium composed of peptone 14 g/l, cysteine 312 mg/l, hydrogen sulfide 312 mg/l, and resazurin 0.1% v/v was added to the fermentation tube at a volume of 7.5 ml. A fecal inoculum mas made from fecal material by mixing it with phosphate-buffered saline at a concentration of 33%. Two milliliters of inoculum were added to the fermentation tube. Afterward, nitrogen was bubbled into the tube until reaching anaerobic conditions (transparent solution as opposed to pink when oxygen is dissolved). After 20 h at 37°C, microbial activity was halted by immersion in ice for 15 min and tubes were centrifuged to collect the supernatant (fraction available for absorption at the large intestine), which was stored at −80°C until further analysis. Blanks carrying water instead of sample were included in the *in vitro* digestion as well as in the *in vitro* fermentation.

### 2.2 DNA extraction, 16S rRNA gene sequencing, and bioinformatic analysis

The solid fractions derived from *in vitro* fermentation were used to obtain the bacterial suspensions, lysed with lysozyme at a final concentration of 0.1 mg/ml. The genomic DNA extraction was then performed with the MagNaPure LC JE379 platform (Roche) and DNA Isolation Kit III (Roche), following the manufacturer’s instructions. DNA was quantified with a Qubit 3.0 Fluorometer (Invitrogen), while agarose gel electrophoresis (0.8% w/v agarose in Tris-borate-EDTA buffer) was used to determine DNA integrity. Finally, the DNA was stored at −20°C until further processing.

The V3-V4 hypervariable region of the 16S rRNA gene was amplified using a template of 12 ng of microbial genomic DNA, following the Illumina protocol for 16S Metagenomic Sequencing Library Preparation. PCR primers were as described by Klindworth *et al.* (2013) with the forward primer (5′-TCGT CGGC AGCG TCAG ATGT GTAT AAGA GACA GCCT ACGG GNGG CWGCA-G3′) and the reverse primer (5′-GTCT CGTG GGCT CGGA GATG TGTA TAAG AGAC AGGA CTAC HVGG GTAT CTAA TCC3′). Primers were fitted with adapter sequences to make them compatible with the Illumina Nextera XT Index kit. Then, amplicon libraries were pooled and sequenced in an Illumina Miseq sequencer in a 2×300 cycles paired-end run (MiSeq Reagent kit v3). The data for the present study were deposited in the European Nucleotide Archive (ENA) at EMBL-EBI under accession number PRJEB51719.

The DADA2 (v1.8.0) package as implemented in R (v3.6.0) was employed for 16S rRNA gene sequence read processing and forward and reverse merging as well as clustering into amplicon sequence variants (ASVs) (Callahan *et al.* 2016). Filtering and trimming parameters were as follows: maxN = 0, maxEE=c(2,5), truncQ = 0, trimLeft=c(17,21), truncLen=c(270,220), and rm.phix=TRUE. A minimum overlap of 15 nucleotides and a maximum mismatch of 1 were required for read merging. Reads were aligned against the human genome (GRCh38.p13) using Bowtie2 (v2.3.5.1) (Langmead and Salzberg 2012) and matches were discarded.

Once the ASV table was obtained, species-level taxonomic identification was assigned to each ASV with DADA2, applying a 100% identity matching between ASVs and the reference sequences in the SILVA138 reference database (Quast *et al.* 2013). Furthermore, the MegaBLAST tool from BLAST v(2.10.0) was used for those ASVs that were identified with DADA2 at genus level but not at species level, requiring at least 97% identity to be species-level assigned. In addition, we only considered ASVs with the same genus assignation from the DADA2 and MegaBLAST methods as well as with a minimum difference of 2% between the first- and second-best matches. Finally, ASVs with total number of counts lower than 10 were removed.

### 2.3 BacArena algorithm

BacArena is an agent-based modeling framework for cellular communities that allows the time simulation of cell growth in an environment called arena of dimensions *nxm* (Bauer *et al.* 2017). This environment is defined by specific geometry and culture medium. BacArena loads different bacterial cells into this arena and simulates their individual growth and reaction fluxes based on genome-scale models and flux balance analysis (FBA) (Orth *et al.* 2010).

In brief, for a genome-scale metabolic model that involves *c* metabolites and *r* reactions, which are integrated in the stoichiometric matrix *S* of dimensions *cxr*, FBA searches for the reaction flux vector *v* of dimensions *rx*1 that maximizes the flux rate through the biomass reaction, called growth rate (*vbio*), (Equation 1), assuming the steady-state condition (Equation 2) and specific reaction flux limitations (Equation 3), namely:

(1) $$\max\;v_{\mathrm{bio}}$$

(2) s.t.  S·v=0

(3) lb ≤ v ≤ ub

where *lb* and *ub* denote the lower and upper bound for reaction fluxes, respectively, which are defined according to the growth medium (nutrient availability) and reaction reversibility in Equation (3).

Importantly, BacArena imposes an upper bound for the biomass production at each time point $v_{\mathrm{bio}}^{\max}$ in the FBA model to guarantee physiologically feasible conditions:

(4) $$v_{\mathrm{bio}}\;\leq \;v_{\mathrm{bio}}^{\max}$$

The biomass generated at a particular time point for a specific individual in the arena B_(t + 1) is updated according to an exponential growth model and the growth rate obtained from FBA $v_{\mathrm{bio}}^{\mathrm{FBA}}$, namely:

(5) $$B_{t+1}{{=B}_{t}{\cdot v}_{\mathrm{bio}}^{\mathrm{FBA}}+B}_{t}$$

Moreover, nutrient level availability for each individual in the arena at each time point is also updated according to the input/output exchange fluxes predicted by FBA and the diffusion of these metabolites across the environment. Here, the diffusion of nutrients was modeled following the Moore neighborhood strategy, one of the options available in BacArena.

In addition, at each time step, BacArena considers specific cell requirements and environmental conditions to trigger specific events such as cell division, lysis, or movement. Moreover, beyond cross-feeding and competitive microbe–microbe interactions, which can be captured by metabolic models, BacArena can consider interactions via the presence of predators which rapidly end up killing the target cell type, typically mediated by specific toxins. However, these interactions are generally unknown and, thus, their use in the modeling of the microbial population dynamics is uncommon. We detail below the extensions to BacArena to consider microbe–microbe and nutrient–microbe interactions obtained from a Bayesian network model.

Finally, for the sake of clarity, note here that each individual in the arena corresponds to a specific microbe in the bacterial community, which obviously requires a different metabolic model. In our case, for the definition of bacterial species and metabolic models in the community, we used AGREDA (Blasco *et al.* 2021), our previously published metabolic reconstruction of the human gut microbiota.

### 2.4 BN-BacArena algorithm

BN-BacArena is an extension of BacArena that incorporates in the native computational framework a Bayesian network model that integrates microbe–microbe and nutrient–microbe interactions. The Bayesian network was built using the relative bacterial abundance and nutrient composition of 55 *in vitro* fermented foods. We trained the network using 80% of the foods (randomly selected). In order to highlight only strong relationships (those resistant to perturbations of the data), we resampled the data using bootstrapping (*n* = 200) and learnt a new network for each sample. Arc strength was calculated as a measure of how often an arc appeared, and deemed significant for those that were present in more than 50% of the networks.

In brief, there are two approaches to learn Bayesian networks from data (Pearl 1988). Constraint-based structure learning algorithms are used to statistically test conditional independencies among triplets of variables from data. The output of this family of algorithms is graphs, mostly directed acyclic graphs (DAGs), which integrate a large percentage (and whenever possible all) of identified conditional independence constraints. Each time the number of variables in the conditioning part of the hypothesis tests goes up, the cardinality of the dataset from which to learn the structure of the model increases considerably, greatly slowing down the search process. Score- and search-based methods are an alternative approach. A score function relative to data measures the goodness of fit of each candidate Bayesian network to the data. The goal is to find a network structure that optimizes the scoring function. These methods usually start from an initial structure, which can be manually defined or randomly generated. Then, best-scored Bayesian networks are proposed using a search method responsible for intelligent movements in the space of possible network structures. There are different space structures, where DAGs are the most used. In the BN-BacArena algorithm, we used this second approach, available in the R package *bnlearn* (Scutari 2010). The scoring function (to be minimized) was the penalized likelihood called Bayesian Information Criterion (BIC) (Schwarz 1978), where the estimated log-likelihood of the data given the Bayesian network adds a term that penalizes the network complexity to avoid structural overfitting. Starting from an empty network, we followed a search process based on the greedy hill-climbing procedure (*hc* function in *bnlearn*), where local changes to the current network (arc addition, arc deletion, arc reversal) are repeatedly applied until there is no further improvement of the BIC score. Note here that *bnlearn* gives the possibility to prevent specific subsets of arcs from appearing in the resulting network (*blacklist* parameter in *hc* function). We forbade nutrient–nutrient and bacteria → nutrient interactions, as they are biologically meaningless, enabling nutrient → bacteria and bacteria–bacteria interactions.

As a result of the score and search process, for each bacterial species node, a linear regression model was obtained using as explanatory variables its associated parent nodes in the Bayesian network: the relative abundance of its parent bacterial species and/or the abundance of its parent nutrients. The resulting models were incorporated into the BacArena algorithm to update species biomass accordingly, as described below. As the nodes are continuous random variables, we assumed they followed the Gaussian probability function. Thus, we can determine the significance of the relationships between microbes and other microbes, as well as microbes and nutrients, through the coefficients of such linear regression model. These coefficients define the relationship between individual variables and their respective parent variables within the Bayesian network. Henceforth, we will refer to $\beta_{i}^{C}$ and $\beta_{i}^{N}$ as the set of coefficients of the linear regression model for each bacterial species *i* in relation to the other bacterial species and nutrients in the culture medium, respectively. We describe below how the linear regression models within the Bayesian network are integrated with BacArena.

Let $x_{it}$ represent the proportion of bacterial species *i* present in the arena at time step *t*, and let $M_{kt}$ represent the molar concentration of each nutrient in the environment at time step *t*. Then, for each bacterial species *i* and time step *t*, we can define a factor $\alpha_{it}\;$as:

(6) $$\alpha_{it}=\frac{x_{it}\;+\;\sum_{j\in C}\beta_{i}^{j}\cdot x_{jt}\;+\;\sum_{k\in N}\beta_{i}^{k}\cdot M_{kt}}{x_{it}}$$

and, accordingly, the upper boundary of the growth rate of every individual of bacterial species *i* in the arena is modified as follows:

(7) $$v_{\mathrm{bio}}\;\leq \;{\alpha_{it}\cdot v}_{\mathrm{bio}}^{\max}$$

Clearly, when $\beta_{i}^{C}=\beta_{i}^{N}=0$, $\alpha_{it}=1$, and, thus, our approach does not have any effect. If $\alpha_{it}\;>\;1$, the microbe–microbe and nutrient–microbe interactions have stimulatory effect on the growth of the bacterial species. The opposite occurs when $\alpha_{it}\;<\;1$.

In summary, the general idea of our approach is to guide the growth of every individual in the arena according to an empirically driven Bayesian network that considers the presence of other bacterial species and nutrients in the environment and modifies the upper bound for the growth rate in the FBA model at each time step. We call our proposed method BN-BacArena.

### 2.5 BN-BacArena validation

For each of the 11 validation set recipes, 20 random computer runs were conducted for both BN-BacArena and BacArena. The random simulations yield distinct cell distributions in the arena and different initial biomass values assigned to every cell. In each of the runs, the time-dependent relative abundance of bacterial species was predicted over 6-time iterations (measured in h). The bacterial species were randomly located in the arena based on 16S rRNA gene sequencing data of the fermented samples. The random initial biomass for each cell was fixed following the standard parameters of BacArena.

### 2.6 Statistical analysis

Relationships between the simulated relative abundance and *in vitro* relative abundance (obtained from16S rRNA gene sequencing data) were performed via Pearson correlation and linear regression. Computation times between MATLAB and R were compared via Wilcoxon test. *P*-values were adjusted for false discovery rate with the Benjamini–Hochberg method (Benjamini and Hochberg 1995). Adjusted *P*-values <.05 were deemed significant. Statistical analysis was performed in R version 4.2.0 and MATLAB version R2018a.

## 3 Results

### 3.1 BacArena implementation into MATLAB

We translated most of the functionalities of the native BacArena code in R to MATLAB, including cell growth, nutrient diffusion, presence of predators, cell movement, or chemotaxis among others (see Supplementary Table S1). All the BacArena objects were integrated into a single structure while maintaining the different relationships between them as in the original code. Moreover, many statements were substituted according to COBRA functions to be amenable and integrable within the COBRA Toolbox software. We also included the associated Bayesian network-based decision step in BN-BacArena in both MATLAB and R.

In order to ensure that the MATLAB version of BacArena and BN-BacArena reached the same simulation results than the R version, we ran 20 simulations for each of the 11 validation foods with the same initial gut microbial composition. For each food, we performed linear regressions between microbial mean relative abundance across replicates obtained using MATLAB and R versions as well as Pearson correlations (Supplementary Fig. S1). Linear regression *P*-values were always significant and lower than 2.2^e-16^, achieving high correlation coefficients ∼1. The same outcome was obtained for BacArena (Supplementary Fig. S2). These results definitely suggest that the translation from R to MATLAB was successful.

We also recorded the computation time for R and MATLAB. MATLAB times were significantly lower (*P *<* *.05) (Supplementary Fig. S3). The main reason of this difference is the computation time for simulating cell growth in the original BacArena code, which grows significantly as the number of bacteria in the arena increases in R in comparison with MATLAB.

### 3.2 Bayesian network interactions

In order to identify bacteria–bacteria (inhibitory or cooperative) and nutrient–bacteria (inhibitory or favorable) interactions, we built a Bayesian network with the strategy described in Section 2. The input data consisted of an *nxP* matrix, where *P =* 216, namely 19 different bacterial species (relative abundance) and 197 different nutrients (expressed in their corresponding units) found in the fermented foods, whereas *n* represented each of the fermented foods (*n *=* *55). The Bayesian network was built using 80% of the samples randomly selected (*n *=* *44). To test the network, bacterial abundance was predicted on the remaining samples (*n *=* *11) and Pearson correlations between predicted values and real values were calculated. We obtained 12 out of 19 significant correlations (*P *<* *.05) with correlation coefficients ranging from 0.67 to 0.96. Overall, the Bayesian network detected 62 significant direct relationships (as determined by arc strength and bootstrapping) (Supplementary Table S2); 23 of those were nutrient–bacteria relationships, which mostly included xanthines, phenolic compounds, polyols, ascorbic acid, and carotenoids.

### 3.3 Bayesian network incorporation into BacArena

As detailed in Section 2, the resulting Bayesian network was introduced into BacArena, leading to BN-BacArena. In particular, with the significant direct relationships obtained from the Bayesian network, we built a linear regression model for each bacterial species and predicted the stimulatory/inhibitory effect of other bacterial species and nutrients for each time step. This step influences the biomass update and other events, including cell death or cell duplication, which are governed by the native BacArena growth function. In order to validate our proposed strategy, we run BN-BacArena using as growth medium input each of the foods that were not used for the Bayesian network training (11 foods). Each of the test foods were run over six-time iterations and, thus, 6 h of simulation. We performed 20 random runs of each case to consider the underlying variability of agent-based modeling. We repeated the same analysis with the native tool, BacArena. The ability of both approaches to predict bacterial growth during *in vitro* fermentations of the 11 validation foods was quantified via the Pearson correlation between the mean simulated relative abundance (across 20 random runs) and the relative abundance obtained from *in vitro* experiments.

**Figure 1.**
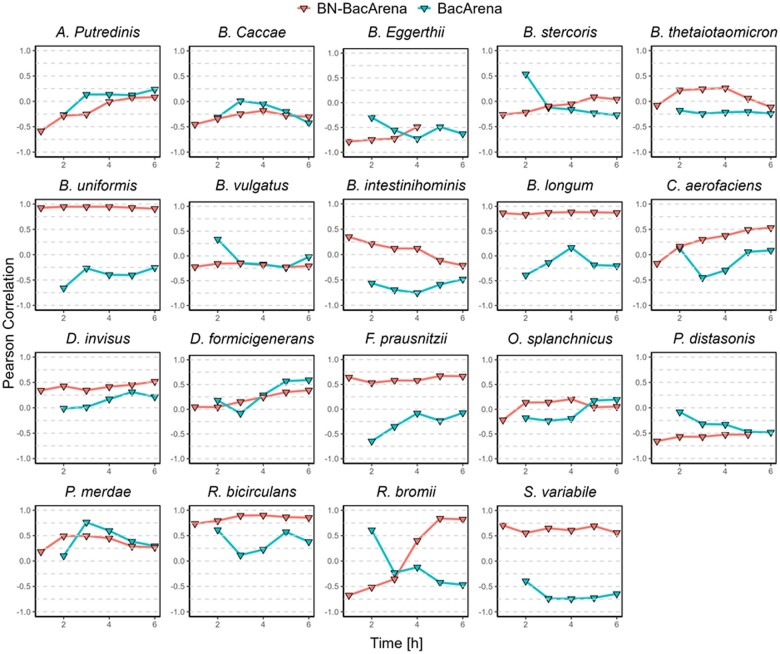
Pearson correlations between simulated relative abundance and *in vitro* relative abundance with BN-BacArena and BacArena. For each species and time point, the mean simulated bacterial relative abundance (across 20 random runs) for the 11 validation foods was correlated with *in vitro* experimental levels after 20 h of fermentation. Abbreviations: *A. putredinis*, *Alistipes putredinis*; *B. caccae*, *Bacteroides caccae*; *B. eggerthii*, *Bacteroides eggerthii*; *B. stercoris*, *Bacteroides stercoris*; *B. thetaiotaomicron*, *Bacteroides thetaiotaomicron*; *B. uniformis*, *Bacteroides uniformis*; *B. vulgatus*, *Bacteroides vulgatus*; *B. intestinihominis*, *Barnesiella intestinihominis*; *B. longum*, *Bifidobacterium longum*; *C. aerofaciens*, *Collinsella aerofaciens*; *D. invisus*, *Dialister invisus*; *D. formicigenerans*, *Dorea formicigenerans*; *F. prausnitzii*, *Faecalibacterium prausnitzii*; *O. splanchnicus*, *Odoribacter splanchnicus*; *P. distasonis*, *Parabacteroides distasonis*; *P. merdae*, *Parabacteroides merdae*; *R. bicirculans*, *Ruminococcus bicirculans*; *R. bromii*, *Ruminococcus bromii*; *S. variabile*, *Subdoligranulum variabile*. See Supplementary Table S3 for more details

As shown in Fig. 1, considering the 19 species that composed the *in silico* microbial community, BN-BacArena was able to simulate significantly better (*P*-value <.05) the growth of nine species (*Bacteroides thetaiotaomicron, Bacteroides uniformis, Barnesiella intestinihominis, Bifidobacterium longum, Collinsella aerofaciens, Dialister invisus, Faecalibacterium prausnitzii, Ruminococcus bicirculans*, and *Subdoligranulum variabil*e, see Supplementary Table S4), whereas the native tool only achieved significantly better results for two species (*Alistipes putredinis* and *Parabacteroides distasonis*).

**Figure 2.**
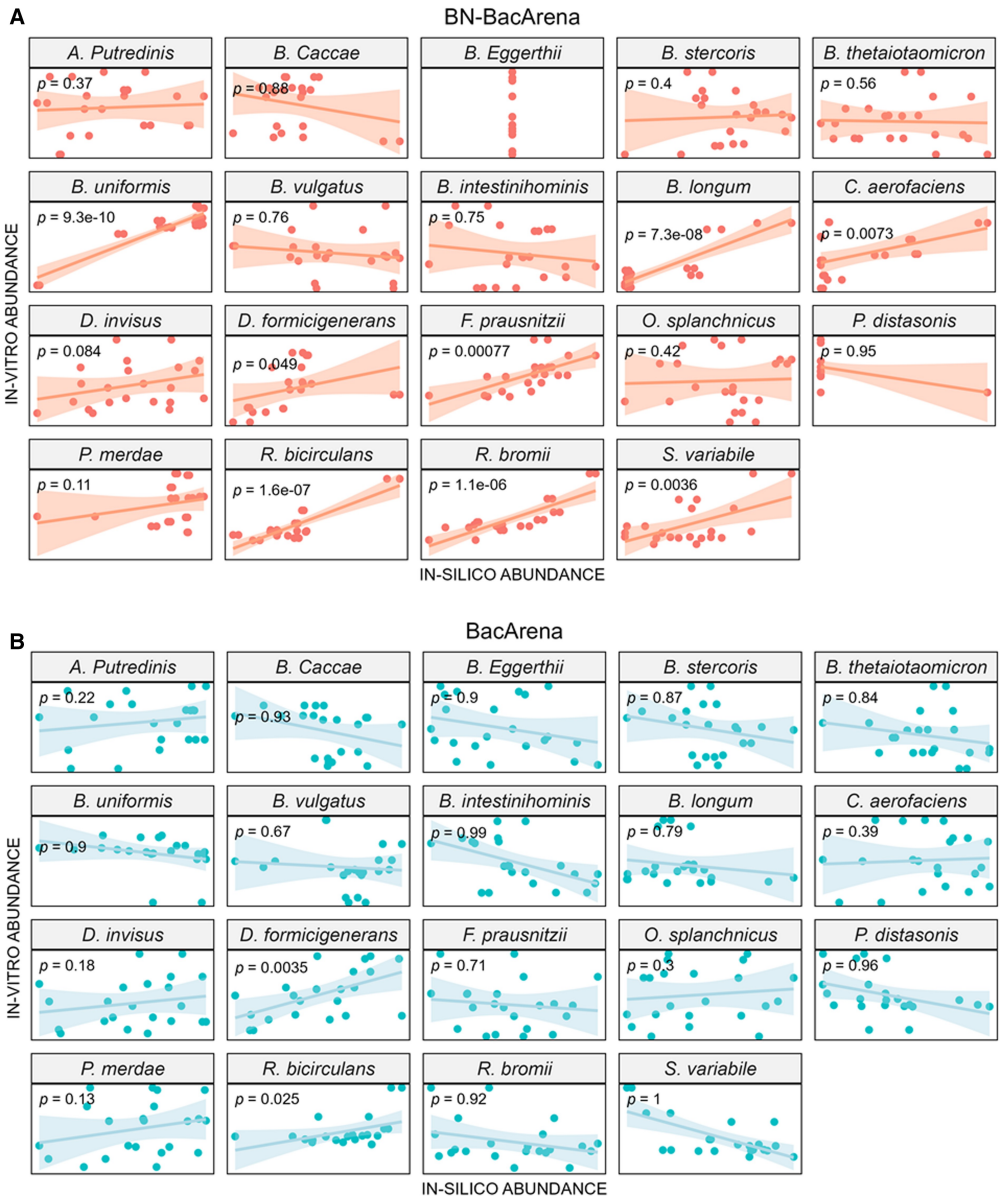
Linear regression between simulated relative abundance and *in vitro* relative abundance with BN-BacArena and BacArena. The linear regression was performed considering simulated bacterial relative abundances across the different foods at 5–6 h and the *in vitro* results. (A) Simulation results using BN-BacArena and (B) BacArena. Abbreviations: *A. putredinis*, *Alistipes putredinis*; *B. caccae*, *Bacteroides caccae*; *B. eggerthii*, *Bacteroides eggerthii*; *B. stercoris*, *Bacteroides stercoris*; *B. thetaiotaomicron*, *Bacteroides thetaiotaomicron*; *B. uniformis*, *Bacteroides uniformis*; *B. vulgatus*, *Bacteroides vulgatus*; *B. intestinihominis*, *Barnesiella intestinihominis*; *B. longum*, *Bifidobacterium longum*; *C. aerofaciens*, *Collinsella aerofaciens*; *D. invisus*, *Dialister invisus*; *D. formicigenerans*, *Dorea formicigenerans*; *F. prausnitzii*, *Faecalibacterium prausnitzii*; *O. splanchnicus*, *Odoribacter splanchnicus*; *P. distasonis*, *Parabacteroides distasonis*; *P. merdae*, *Parabacteroides merdae*; *R. bicirculans*, Ruminococcus bicirculans; *R. bromii*, *Ruminococcus bromii*; *S. variabile*, *Subdoligranulum variabile*

Additionally, we performed linear regression between the simulated relative abundances and *in vitro* relative abundances for both approaches. In this case, we used mean relative abundances of those 20 runs and only the relative abundances obtained in times 5 and 6, when the simulation has reached a stable community. In this case, the incorporation of the Bayesian network resulted in significant linear relationships for *Bifidobacterium longum, Collinsella aerofaciens, Dialister invisus, Dorea formicigenerans, Faecalibacterium prausnitzii, Ruminococcus bicirculans, Ruminococcus bromii*, and *Subdoligranulum variabile*, whereas the use of the native tool showed just two biologically relevant linear relationships (*Bacteroides caccae* and *Ruminococcus bicirculans*) (Fig. 2A and B). The most accurate results were obtained with *Bacteroides uniformis* (S6) and *Bifidobacterium longum* (S9) in the case of BN-BacArena, where we found correlations near 1 (Fig. 1). Therefore, we concluded that the incorporation of the Bayesian network information to BacArena significantly improves the predictive capacity.

### 3.4 Metabolic interactions in BN-BacArena and BacArena

As noted above, BacArena can account for bacteria–bacteria interactions at the metabolic level, such as cross-feeding interactions and nutrient competition interactions. However, other bacteria–bacteria interactions that go beyond genome-scale metabolic modeling are also important but typically unknown. With our Bayesian network model, we directly address this limitation of existing tools and predict undocumented bacterial relationships. Here, we aim to explore further the set of predicted interactions in the Bayesian network model, which can be either positive or negative, particularly their relevance at the metabolic level in both BN-BacArena and BacArena.

**Figure 3.**
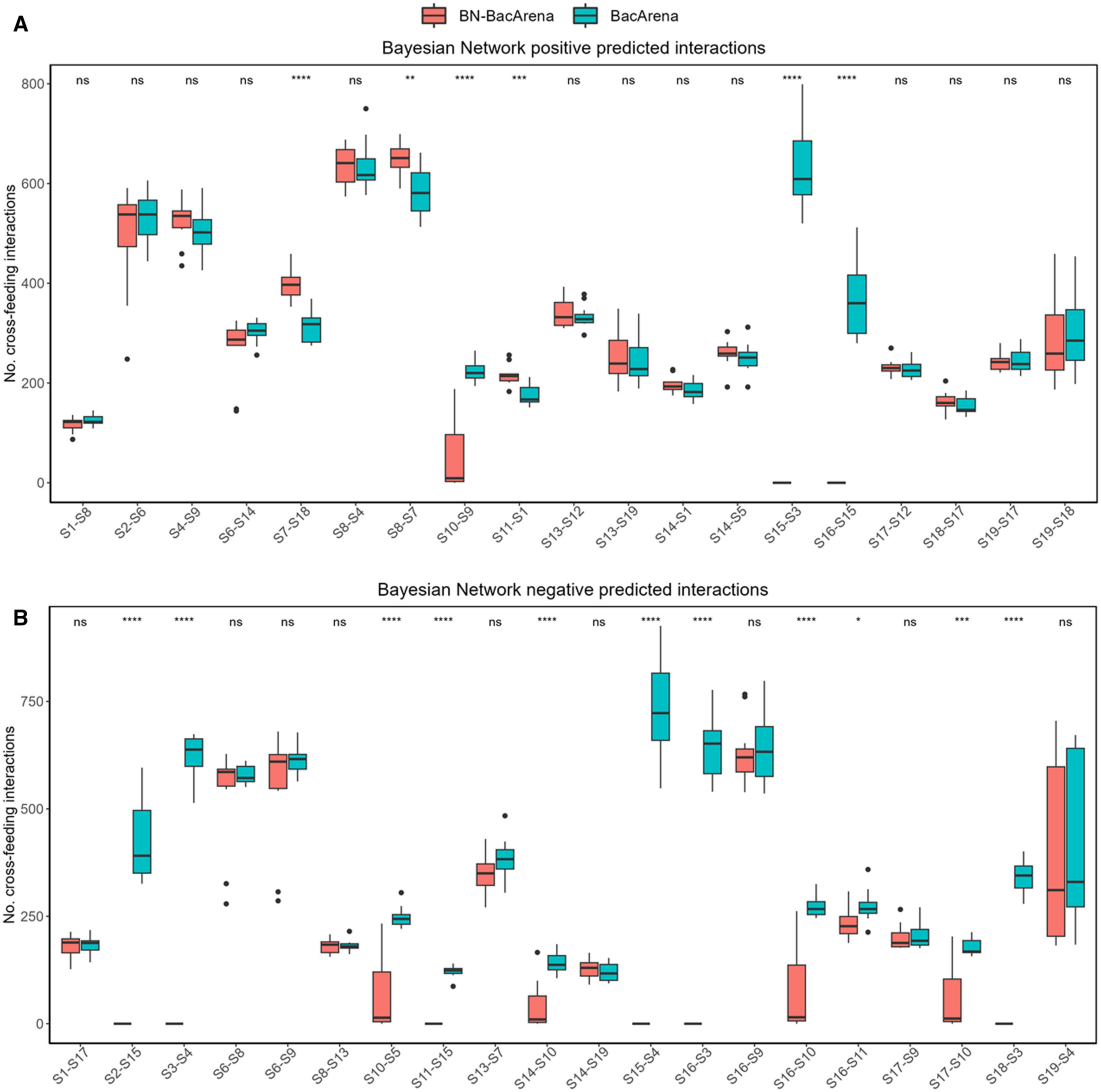
Cross-feeding interactions in BN-BacArena and BacArena for bacteria–bacteria relationships in the Bayesian network model. The cross-feeding interactions were estimated at the end of the simulations across the different foods and replicates. Number of cross-feeding interactions for the 19 predicted positive (A) and 20 negative (B) microbe–microbe interactions in the Bayesian network model. Note: ‘S1-S8’, for example, means that S1 regulates S8. Thus, a cross-feeding interaction implies that S1 produces an output metabolite that is received by S8. Abbreviations: S1, *Alistipes putredinis*; S2, *Bacteroides caccae*; S3, *Bacteroides eggerthii*; S4, *Bacteroides stercoris*; S5, *Bacteroides thetaiotaomicron*; S6, *Bacteroides_uniformis*; S7, *Bacteroides_vulgatus*; S8, *Barnesiella_intestinihominis*; S9, *Bifidobacterium longum*; S10, *Collinsella aerofaciens*; S11, *Dialister invisus*; S12, *Dorea formicigenerans*; S13, *Faecalibacterium prausnitzii*; S14, *Odoribacter splanchnicus*; S15, *Parabacteroides distasonis*; S16, *Parabacteroides merdae*; S17, *Ruminococcus bicirculans*; S18, *Ruminococcus bromii*; S19, *Subdoligranulum variabile*

To that end, for each microbial pairwise interaction predicted by the Bayesian network, we determined the number of cross-feeding interactions occurring with the different foods and replicates both in BacArena and in BN-BacArena. Note here that cross-feeding interactions were identified as in BacArena, namely identifying if two bacteria share exchange fluxes, i.e. an output metabolite in one microbe is used as input metabolite by another microbe. Moreover, based on the linear regression coefficients provided by the Bayesian network, we classified each microbial pairwise interaction as positive or negative, i.e. mutualistic or competitive interactions. Full results are shown in Fig. 3.

As shown in Fig. 3A, both BN-BacArena and BacArena show similar results for predicted positive interactions in the Bayesian network model, specifically each of them found significantly higher cross-feeding interactions in 3 cases out of 19. However, when considering predicted negative interactions, BN-BacArena successfully predicts a lower number of interactions for 11 cases in comparison with BacArena. The same result was found in both MATLAB and R (see Supplementary Fig. S4). Hence, we can conclude that BN-BacArena alters the structure of metabolic interactions with respect to BacArena.

## 4 Discussion

There are several relevant computational tools for modeling gut microbial metabolism (Magnúsdóttir *et al.* 2016, Blasco *et al.* 2021). However, the prediction of bacterial dynamics in the gut microbiota is still a challenging task, particularly given that external factors, such as diet, are highly dynamic. Although BacArena and other tools provide this functionality, they still lack the ability to comprehensively account for bacteria–bacteria stimulant or inhibitory effects or nutrient–bacteria inhibitory effects, such as those exhibited by some phenolic compounds (Monlau *et al.* 2014).

Here, we present an update of BacArena, called BN-BacArena, consisting on the incorporation within the algorithm of a Bayesian network model that integrates bacteria–bacteria and nutrient–bacteria interactions. The Bayesian network showed 23 significant nutrient–bacteria interactions, suggesting that compounds such as polyols, ascorbic acid, polyphenols, and other phytochemicals can mediate bacterial growth either directly or indirectly by affecting competitors or cooperators. Relationships between bacterial species have been previously described, especially for fiber degradation where a first degrader establishes itself at the fiber surface and starts breaking down the structure releasing smaller compounds that are then used by other species (Trosvik and de Muinck 2015, Cann *et al.* 2016). However, considering the complexity and diversity of gut ecology, undocumented relationships are still bound to happen. Our Bayesian network detected 40 bacteria–bacteria significant relationships.

We used a Gaussian Bayesian network model to extract bacteria–bacteria and nutrient–bacteria interactions and, thus, data normality is assumed. This assumption is not ideal for (compositional) relative abundance data; however, it substantially simplifies the analysis and interpretation of the resulting model. We expect to refine our approach in the future using nonparametric Bayesian network models currently under development.

According to our results, BN-BacArena improved bacterial growth simulations on different foods as showed by Pearson correlations and linear regression models. More specifically, Pearson correlations obtained between simulated and experimental values were higher for nine bacterial species (out of 19) when compared to the native tool. On the other hand, the native tool achieved better correlations for two species. Additionally, we obtained eight significant (*P *<* *.05) linear relationships when using Bayesian network information and only two when using the native tool. Importantly, we showed that BN-BacArena changed the landscape of bacteria–bacteria metabolic interactions through the different simulations with respect to BacArena, according to the predicted interactions in the Bayesian network model.

Interestingly, it can be noted that the Bayesian network model obtains better predictions of relative abundance than BN-BacArena or BacArena. However, agent-based modeling approaches and constraint-based modeling allow us to more globally analyze the performance of bacterial communities at the metabolic level. In particular, as done in previous works in the literature (Blasco *et al.* 2021, Balzerani *et al.* 2022), BN-BacArena can be applied to predict the production of output microbial metabolites of interest, such as short-chain fatty acids, which cannot be done with our current Bayesian network model. Future work will include applying and assessing the performance of BN-BacArena to predict the production of key metabolites in the human gut microbiota under different scenarios.

Finally, we have proven that using experimental data to update existing algorithms can improve their simulating abilities. Next steps should include, on one hand, experimentally validating the relationships obtained via pure bacterial culture and co-cultures and, on the second hand, scaling-up the Bayesian network input including data from big human cohorts with thorough nutritional information, in order to build an algorithm that can be applied for the general population or for those suffering from specific illnesses such as inflammatory bowel disease, obesity, etc.

## Supplementary Material

btae266_Supplementary_Data

## Data Availability

MATLAB and R code are available in https://github.com/PlanesLab/BN-BacArena. The other data underlying this article will be shared on reasonable request to the corresponding author.
