# Supplementary material for: BN-BacArena: Bayesian network extension of BacArena for the dynamic simulation of microbial communities
Source: Bioinformatics. 2024 Apr 30;40(5):btae266. doi: 10.1093/bioinformatics/btae266 (PMC11082422; doi:10.1093/bioinformatics/btae266)
Supplement: btae266_Supplementary_Data [file btae266_supplementary_data.zip › Supplementary Information.docx]

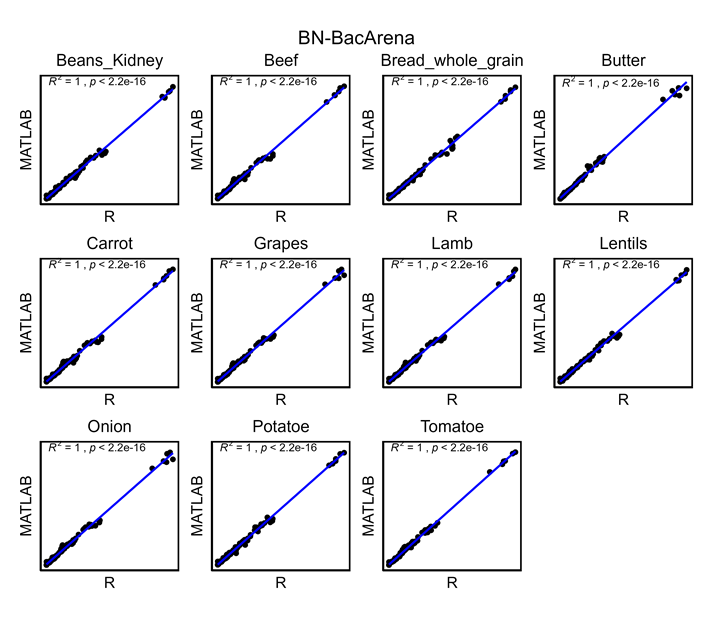


**Supplementary Figure 1. Comparison between R and MATLAB simulations for BN-BacArena.** Correlation of the predicted relative abundance of each bacterial species among 11 validation foods in BN-BacArena. The expected relative abundance of each cell type was compared between the MATLAB and R approaches of BN-BacArena.


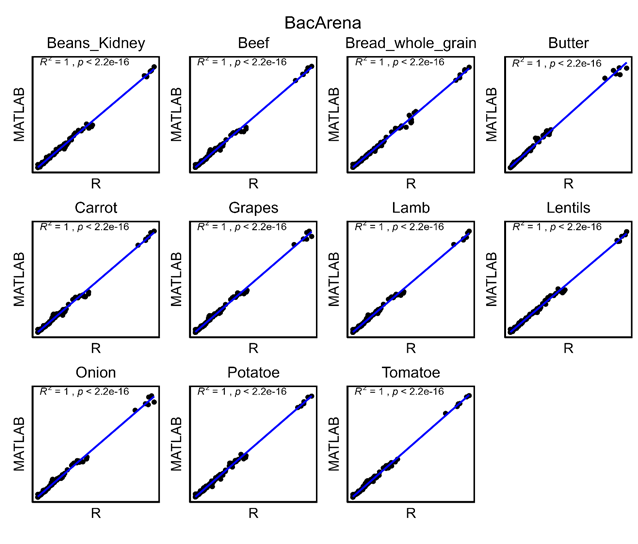


**Supplementary Figure 2. Comparison between R and MATLAB simulations for BacArena.** Correlation of the predicted relative abundance of each bacterial species among 11 validation foods in BacArena. The expected relative abundance of each cell type was compared between the MATLAB and R approaches of BacArena.


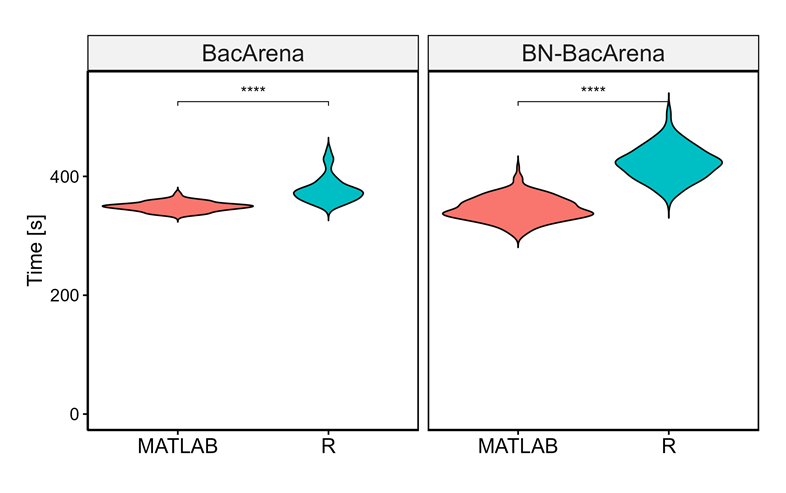


**Supplementary Figure 3. Computation time for both Bac-Arena and BN-BacArena in 20 random simulations.** Comparisons were made via Wilcoxon test. Statistical labels: ****: p<0.0001.


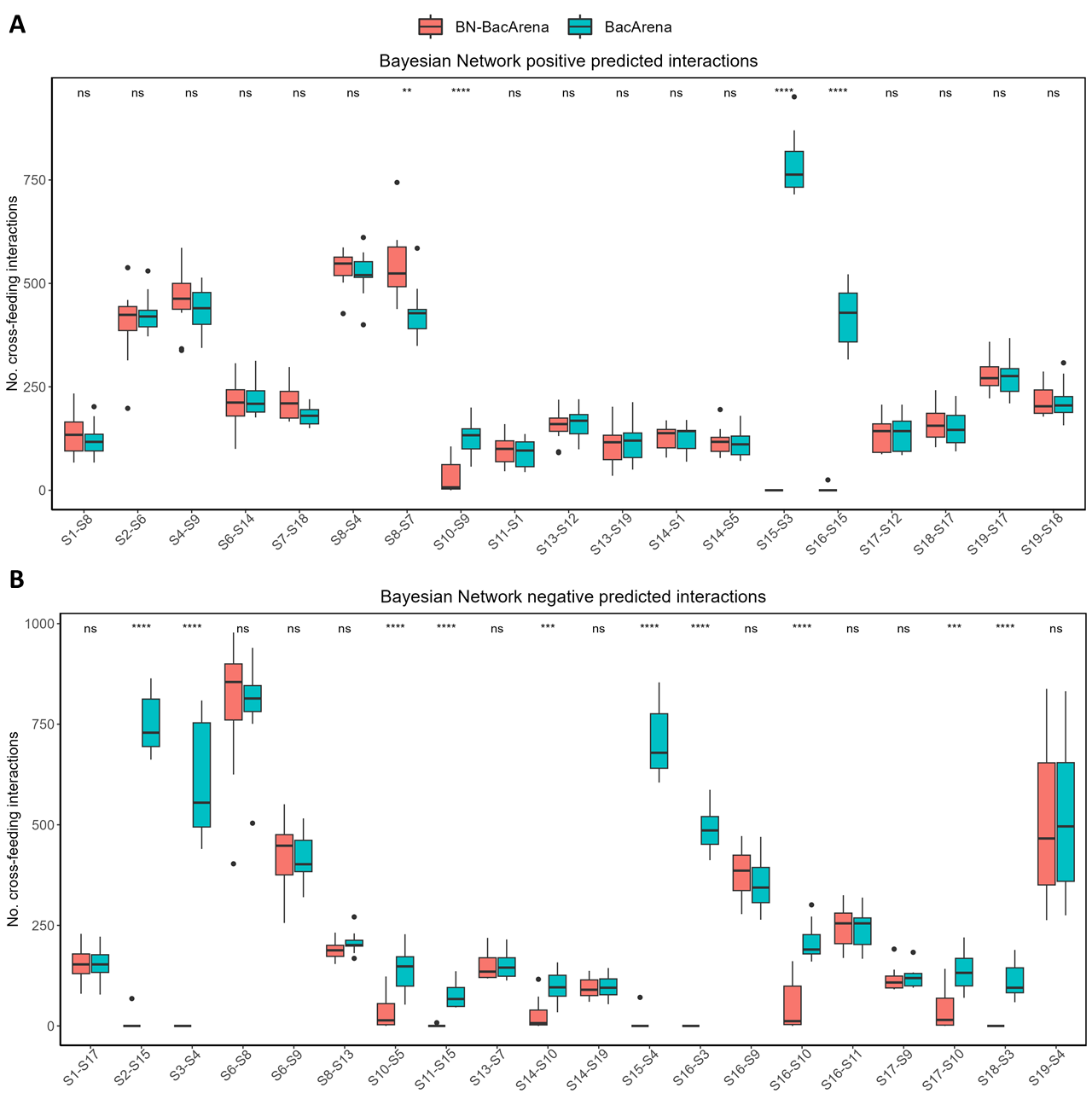


**Supplementary Figure 4**. **Cross-feeding interactions in BN-BacArena and BacArena for bacteria-bacteria relationships in the Bayesian network model with MATLAB**. The cross-feeding interactions were estimated at the end of the simulations across the different foods and replicates. Cross-feeding interactions for the predicted positive (**A**) and negative (**B**) microbe-microbe interactions in the Bayesian network model. Note: ‘S1-S8’, for example, means that S8 regulates S1. Thus, a cross-feeding interaction implies that S8 produces an output metabolite that is received by S1. Abbreviations: S1: *Alistipes putredinis*; S2: *Bacteroides caccae*; S3: *Bacteroides eggerthii*; S4: *Bacteroides stercoris*; S5: *Bacteroides thetaiotaomicron*; S6: *Bacteroides_uniformis*; S7: *Bacteroides_vulgatus*; S8: *Barnesiella_intestinihominis*; S9: *Bifidobacterium longum*; S10: *Collinsella aerofaciens*; S11: *Dialister invisus*; S12: *Dorea formicigenerans*; S13: *Faecalibacterium prausnitzii*; S14: *Odoribacter splanchnicus*; S15: *Parabacteroides distasonis*; S16: *Parabacteroides merdae*; S17: *Ruminococcus bicirculans*; S18: *Ruminococcus bromii*; S19: *Subdoligranulum variabile.*
